# The Hidden Bacterial World of Natural Springs: Insight Through MALDI‐TOF‐Mass Spectrometry

**DOI:** 10.1002/mbo3.70021

**Published:** 2025-10-29

**Authors:** Rizwan Abbas, Muhammad Haris, Sidra Rahman, Syed Ahsan Shahid, Tasbiha Gul, Ayesha Farooq, Alaa Ismail, Modi O. Alotaibi, Gaber El‐Saber Batiha, Muhammad Ali

**Affiliations:** ^1^ Department of Biotechnology Quaid‐i‐Azam University Islamabad Islamabad Pakistan; ^2^ Natural and Medical Sciences Research Center University of Nizwa Nizwa Oman; ^3^ Microbiology and Immunology Department University of Otago Dunedin New Zealand; ^4^ Public Health Laboratories Division National Institute of Health Islamabad Pakistan; ^5^ School of Medicine Taif University Taif Saudi Arabia; ^6^ Department of Biology, College of Science Princess Nourah bint Abdulrahman University Riyadh Saudi Arabia; ^7^ Environmental and Biomaterial Unit, Natural and Health Sciences Research Center Princess Nourah bint Abdulrahman University Riyadh Saudi Arabia; ^8^ Department of Pharmacology and Therapeutics Faculty of Veterinary Medicine, Damanhour University Damanhour AlBeheira Egypt

**Keywords:** freshwater microbial diversity, Gilgit Baltistan, MALDI‐TOF‐MS, natural springs, opportunistic pathogens, Shigar

## Abstract

This study explored the microbial diversity in two underexplored natural springs, *Arincho Chumik* and *Chutron*, located in the Shigar Valley, Gilgit Baltistan, utilizing a culture‐centered method combined with Matrix‐Assisted Laser Desorption Ionization‐Time of Flight Mass Spectrometry (MALDI‐TOF MS). The outcomes revealed a diverse microbial landscape, with a total of 18 unique bacterial strains isolated, comprising nine from each spring. From the total 18 isolated strains, 7 (39%) were noticed to be gram‐positive, while 11 (61%) were gram‐negative. Interestingly, species like *Brevundimonas* and *Acinetobacter* were present in *Arincho* and *Chutron* springs, respectively, highlighting the unique physicochemical environments and their impact on microbial populations. The examination also uncovers the existence of pigment‐producing bacteria, suggesting potential biotechnological applications. The chilly freshwater spring of *Arincho* possessed certain opportunistic bacteria, including *Bacillus cereus, Dietzia cinnamea*, and *Microbacterium* species. Likewise, human‐related microorganisms like *Micrococcus leuteus* were also identified in samples from the *Chutron* thermal spring. Additionally, the recognition of opportunistic pathogens among the strains underlines the health effects for the local communities, especially for the elderly and immune‐deficient individuals. The quality of these water resources ought to be supervised by regulatory authorities to decrease public health risks and pathogen transmission.

## Graphical abstract

MALDI‐TOF MS reveals diverse bacterial populations in Shigar Valley's natural springs, including opportunistic pathogens and pigment‐producing species. This study highlights the need for monitoring water quality to mitigate public health risks and explores potential biotechnological applications.

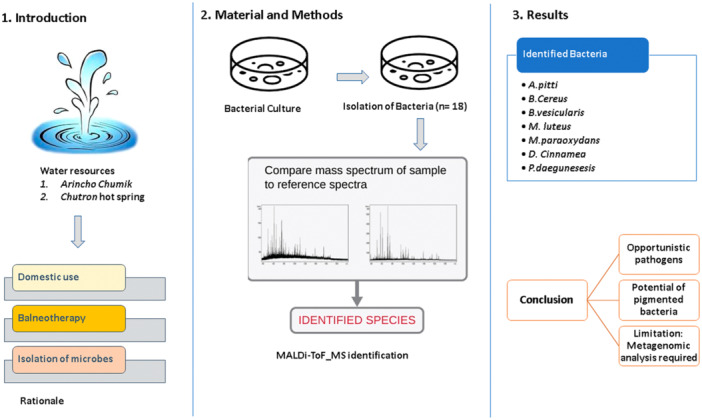

## 1 Introduction

Underground water sources are essential ecosystems that nurture a variety of microorganisms, serving as vital habitats for their development. These include mineral springs, aquifers, and particularly geothermal springs, which are known for supporting life forms such as thermophiles (Aanniz et al. 2015). Such unique characteristics have encouraged microbiologists globally to study the microbial diversity present in geothermal springs (Rehman et al. 2018). Since the first research published by Miquel in 1888, there has been a consistent interest in the study of thermophiles sourced from an array of thermal waters (Adiguzel et al. 2009).

The bacterial communities present in these springs play a crucial role in the biogeochemical process and the sustainability of the underground environment. Beyond thermophiles, both mesophiles and psychrophiles have been recognized as valuable sources of bioactive compounds (Kambourova 2018). Bacterial strains such as *Paraclostridium benzoelyticum, Bacillus subtilis*, and *Bacillus tequilensis* have been identified in spring waters for their ability to synthesize enzymes such as amylase, which hold significant industrial importance (Afridi et al. 2020). Moreover, some microbes may inhibit the growth of environmental contaminants and fecal coliforms, thereby purifying the water (Hutchinson et al. 1943). Nonetheless, the same environment can also support the proliferation of harmful pathogenic bacteria. Previous studies documented the presence of various pathogenic and opportunistic bacteria in water springs. *Clostridium, Pseudomonas, Burkholderia, Aeromonas, Bacillus*, and fecal coliforms including *E. coli* are some of the common examples of water‐borne pathogens (Batool 2018).

In the district setting of Shigar Valley, which is located in Gilgit Baltistan, Pakistan, spring water serves as a crucial resource. Here, the pristine nature of spring water, emerging from glacial and snow runoff, provides the local communities with safe drinking water and supports their daily needs. Additionally, the tradition of balneotherapy, or therapeutic bathing in thermal springs, is prevalent among the locals, a practice supported by scientific evidence for its health benefits (Farhat et al. 2021).

Despite the apparent purity, the microbiological profile of these springs remains largely unexplored, posing potential risks due to the presence of microorganisms and pollutants. It is crucial to check these water resources to ensure safety protocols for residents, as pathogenic microbes may lead to waterborne diseases. Recognizing the health implications, this study aims to scrutinize the water samples from two particular springs: *Arincho Chumik*, known for its refreshing cold water, and Chutron Hot Spring, recognized for its thermal waters.

Identification and characterization of microbes can be done based on phenotypic characteristics, biochemical properties, and genomic sequences through different approaches. The study employs matrix‐assisted laser desorption ionization‐time of flight mass spectrometry (MALDI‐TOF MS) to systematically catalog and identify the bacterial diversity present, ensuring water safety for the surrounding communities.

## 2 Materials and Methods

### 2.1 Study Design

**Figure 1:**
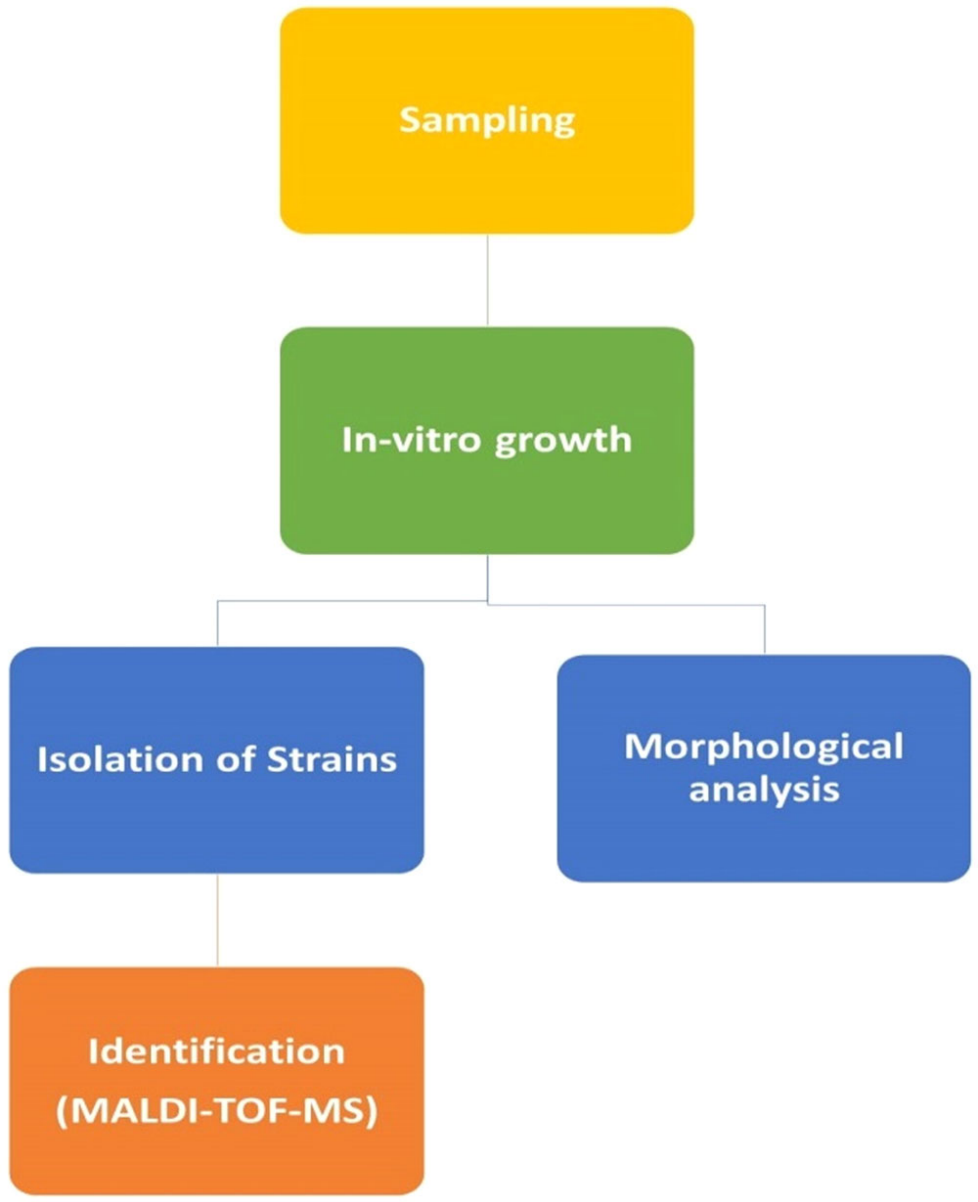
The entire work plan for the study.

This study was performed for the exploration of microbial diversity within two unexplored springs: *Arincho Chumik* (known for its chilly waters) and *Chutron* (characterized by its thermal waters), located in the Shigar Skardu district of Gilgit Baltistan. This work was utilized a culture‐dependent approach to identify the bacterial populations residing in these unique aquatic environments (Figure 1).

### 2.2 Sample Collection

Water samples were collected from *Arincho Chumik* and *Chutron* hot springs in August 2021. The samples were collected by using sterile polyethylene bottles with screw caps to ensure sample integrity and these samples were immediately transported under controlled conditions to the Infectious Diseases and Molecular Pathology Laboratory (IDMPL), Department of Biotechnology, Quaid‐i‐Azam University, Islamabad. Physical properties were also observed during sample collection, and strict practices were undertaken for sample handling, transportation, and preservation to prevent any contamination or alteration of the samples.

### 2.3 Bacterial Colonies Isolation and Purification

Water samples were inoculated on Tryptic Soya Agar (TSA) medium, and different strains of Bacteria were isolated and purified by using the streaking method. For this purpose, inoculated TSA plates were incubated at varying temperatures (10°C and 37°C) for 48 h. Plates were observed periodically for bacterial growth and different colonies were subcultured on base of morphological features such as color, shape, texture, margin and surface etc. After isolation and purification, Gram staining was performed according to the standard protocol.

### 2.4 Identification and Analysis

The identification of microbial strains was conducted utilizing the Matrix‐Assisted Laser Desorption Ionization‐Time of Flight Mass Spectrometry (MALDI‐TOF MS) technology. The analysis was done at the Microbiology Department of the Public Health Laboratories Division (PHLD), National Institute of Health (NIH), Islamabad.

**Figure 2:**
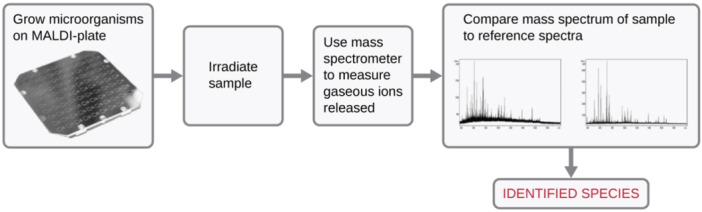
MALDI‐TOF‐MS working principle adopted from Hou et al. (2019).

The colonies that were isolated from the specimens were smeared along with *E. coli* ATCC 8739 on a matrix of coded cards supplied by Biomerieux. For sample preparation, 1 µL of α‐cyano‐4‐hydroxycinnamic acid (CHCA), a matrix substance, was applied to each bacterial smear. The prepared samples were processed for MALDI‐TOF MS analysis, and the data acquired from this process were then cross‐referenced with the MYLA software's library, version 3.1, for strain identification and verification. The working principle of MALDI‐TOF‐MS is represented in Figure 2.

## 3 Results

**Figure 3:**
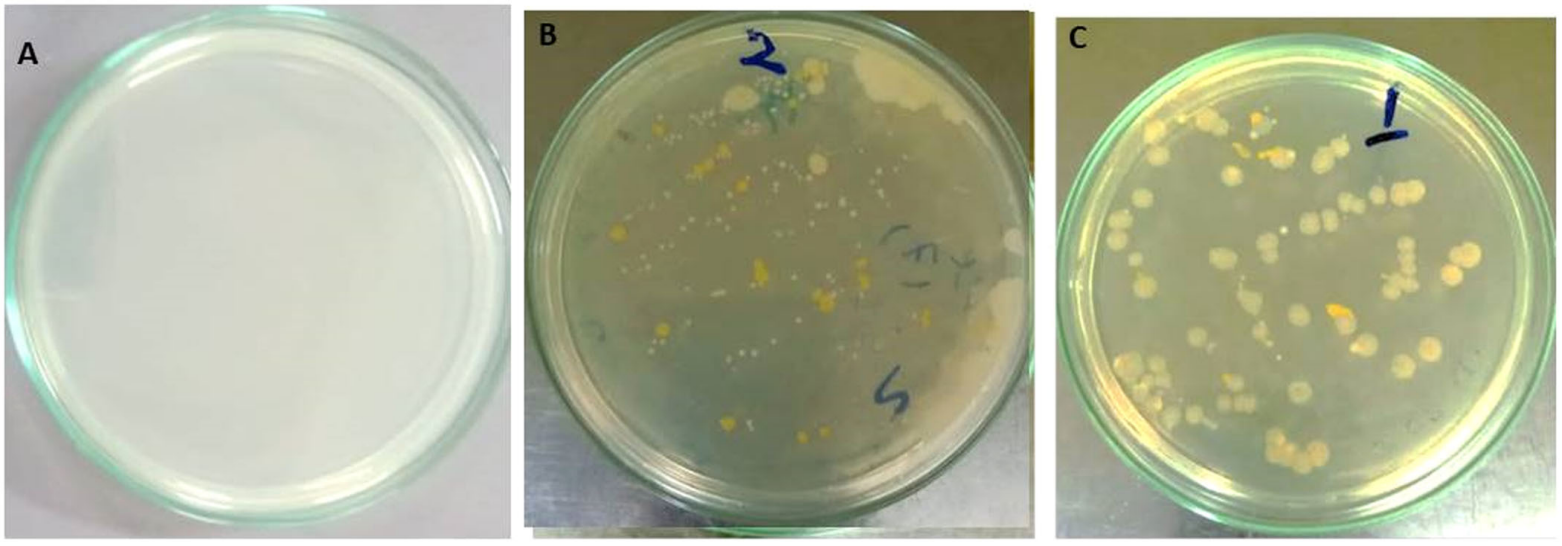
Initial growth observations. (A) No growth at 10, (B and C) considerable growth at 37°C.

We identified the bacterial diversity of *Arincho Chumik* (cold) and *Chutron* (hot) springs located at Shigar Valley Skardu, Gilgit Baltistan. The detailed characteristics of samples are listed in Table 1. Most strains showed considerable growth in the first 24 h (Figure 3).

**Table 1:** Profile of springs and initial observation.

Name	Arincho Chumik	Chutron hot spring
Literal meaning	Water origin/spring	Hot water
Temperature	10°C–20°C	37°C–42°C
pH	6.8–7.0	7.2–7.3
Usage	Domestic	Balneotherapy
Latitude and longitude	36°40′13″ N;	36°40′33″ N;
	75°24′12″ E;	75°26′26″ E;
Location	Shigar, Gilgit Baltistan	
Elevation	8023 ft	

### 3.1 Isolation of Pure Colonies

**Figure 4:**
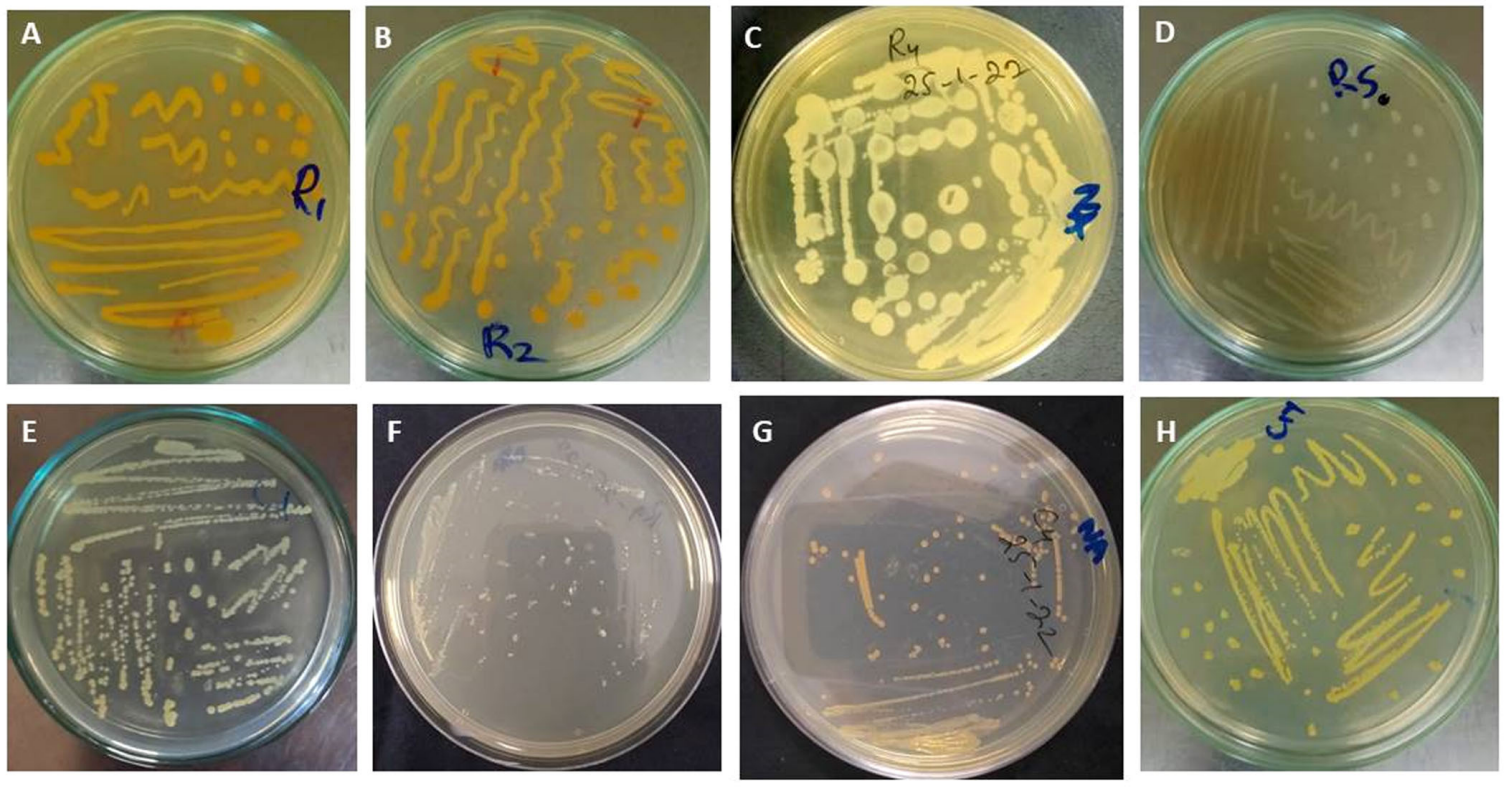
Representation of bacterial isolates grown at 37°C. (A) *M. Paraoxydans*, (B) *M. testaceum*, (C) *B. cereus*, (D) *B. vesicularis*, (E) *A. Pitti*, (F) *A. lacutae*, (G) not identified, (H) *M. Leuteus*.

In this analysis, we successfully isolated and identified a total of 18 (*n *= 18) pure bacterial isolates from the two springs, with nine (*n* = 9) distinct strains being identified from each location. Notably, seven (*n *= 7) of these isolates were found to be pigment‐producing bacteria (Figure 4).

### 3.2 MALDI‐ToF‐MS

**Figure 5:**
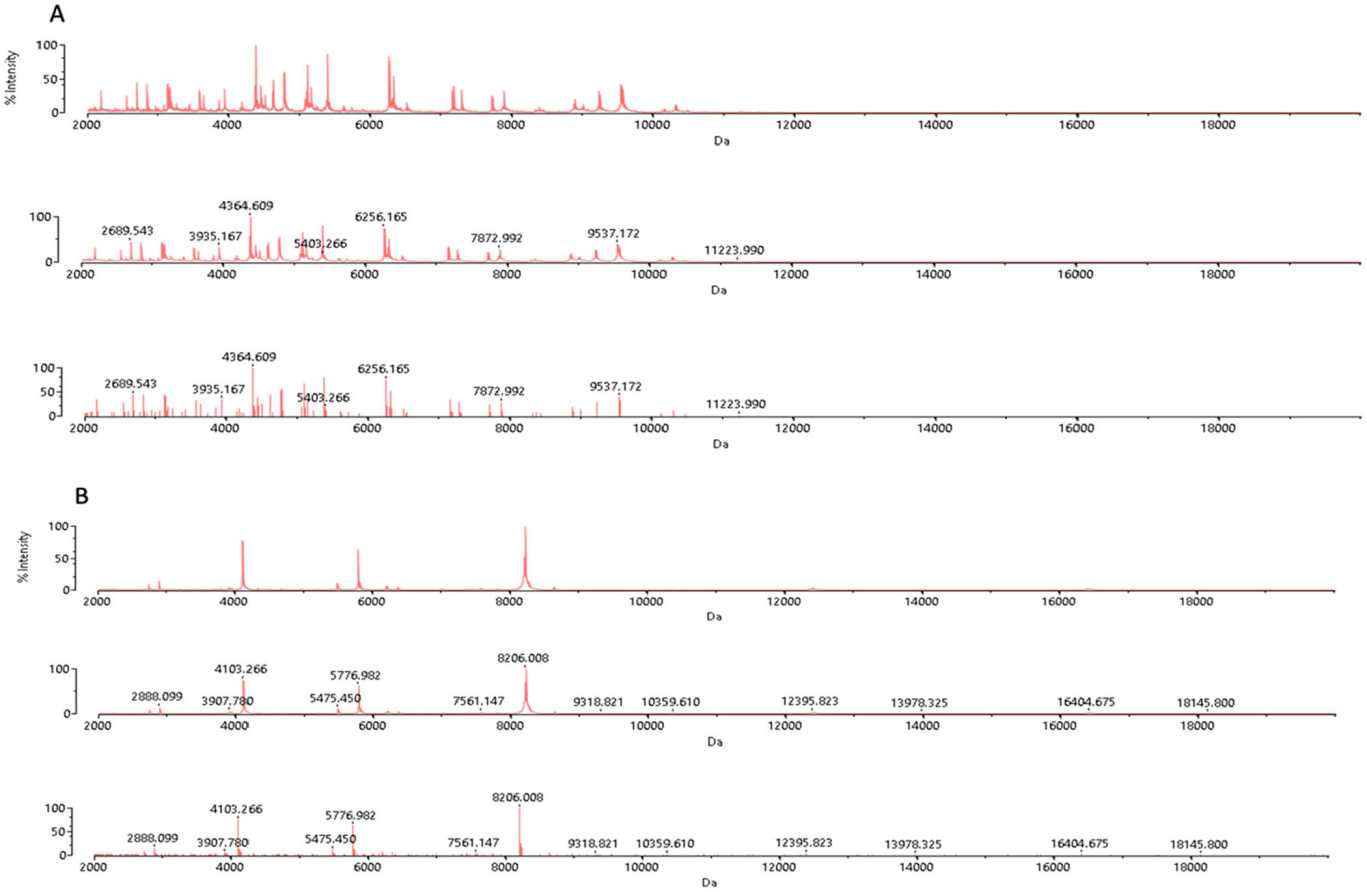
Representation of spectra obtained in MALD‐ToF‐MS analysis: (A) Reference strain *E. coli* ATCC 8739, (B) *A. Pitti*.

It was found that the *Brevundimona*s species dominated the *Arincho* spring, known for its cooler temperatures, while *Acinetobacter* species dominated the *Chutron* hot spring. MALDI‐ToF‐MS based spectral lines are depicted (Figure 5).

The predominant species identified in the *Arincho* spring was *Brevundimonas*, alongside other significant findings, including *Microbacterium paraoxydans, Microbacterium testaceum, Bacillus cereus*, and various *Brevundimonas* species such as *B. vesicularis, B. leutea*, and *B. albigilva*. Additionally, we isolated strains like *B. nasdae* and *Dietzia cinnamea*, showcasing the rich biodiversity present in the cold‐water ecosystem. Conversely, the warmer *Chutron* spring was inhabited by various by *A. pitti, A. leutea, A. calcoaceticus*, and *A. lactucae*. The presence of *Pigmentiphaga daeguensis* and *Micrococcus leuteus* was also noteworthy, underlining the unique thermal adaptations of these organisms. A total of *n* = 4 strains were not identified through MALDI‐TOF MS (Table 2).

**Table 2:** Identification of isolated bacterial strains.

Isolates recovered from Arincho Chumik					
Isolates	Color	Morphology of the colony	Gram staining	Identification	Confidence score
R1	Yellow	Round	GPC	*M. paraoxydans*	99.9%
R2	Yellow	Zigzag	GPC	*M. testaceum*	99.9%
R3	Light yellow	Ovoid	GPC	Not identified	—
R4	White	Swarming	Gram positive bacilli	*Bacillus cereus*	99.9%
R5	Transparent	Round	GNR	*Brevundimonas, vesicularis*	99.9%
R6	Whitish	Dotted	GNR	*B. albigilva*	99.9%
R7	Transparent	Irregular	GNR	*B. leutea*	99.9%
R8	Orange	Round	GPC	*Deitzia cinnamea*	99.9%
R9	Transparent	Irregular	GNR	B. *nasdae*	99.9%

Isolates recovered from Chutron hot spring					
Isolates	Color	Morphology of the colony	Gram staining	Identification	
C1	Creamy	Pointed	GNR	*Acinetobacter pitti*	99.9%
C2	Oily	Dotted	GNR	Not identified	—
C3	Transparent	Zigzag	GNR	*Pigmentiphaga daeguensis*	99.9%
C4	Brownish	Round	GNR	Not identified	—
C5	Light brown	Round	GNR	*A. lactucae*	99.9%
C6	Transparent	Irregular	GNR	Not identified	—
C7	Creamy	Round	GNR	*A. calcoaceticus*	99.9%
C8	Yellow	Round	GPC	*Micrococcus leuteus*	99.9%
C9	Light yellow	Round	GPC	Not identified	—

Abbreviations: GNR, gram‐negative rods; GPC, gram‐positive cocci.

## 4 Discussion

To the best of our knowledge, this study explored the bacterial diversity of springs in Shigar, Gilgit Baltistan, through a culture‐based approach for the first time. In this study, *n* = 18 bacterial strains, nine from each spring were recovered. Actinobacteria were found to be abundant in the cold‐water spring of *Arincho*, while *Proteobacteria* were prominent in the *Chutron* hot spring, which resembles previously reported studies (Amin et al. 2017). The dominance of these diverse microbes could be due to the physiochemical features of spring. Recent studies reported that geochemistry and abiotic factors such as pH, temperature, and mineral content define the microbial diversity in a particular environment (Fariq et al. 2021).

Most strains showed considerable growth at 25°C–37°C during the first 24 h. However, few strains showed growth after 2–4 days of incubation. It could be due to other environmental and physicochemical factors or a slow growth rate. Previous studies support our findings that certain environmental bacteria are tedious to grow under laboratory conditions and could take up to 15 days for growth (Herman 1978). All natural resources, including springs, provide a habitat for diverse microorganisms. Besides, these anthropogenic activities contaminate these resources. Our findings are consistent with previous studies that reported the identification and presence of *Brevundimonas*, *Microbacterium*, *Pigmantiphaga* species in different water resources, including lakes and wastewater (Rao and Kumar 2014; Valcheva et al. 2020). Contradictory to our results, one group reported that *M. testaceum* is an endophytic bacterium (Wang et al. 2010). It might be transmitted from plant roots or other parts found near the spring origin, or anthropogenic activities. Similarly, another study reported the isolation of a novel *B. albigilva* strain from forest soil (Hong et al. 2016). In the current study, *B. albigilva* might adopt a water environment from soil sediments.

We have confirmed the presence of certain opportunistic pathogens including *Acinetobacter* species, *B. cereus*, *D. cinnamea*, *Brevundimona*s species, *M. leuteus*, and other bacteria. Such opportunistic pathogens make these water resources unsuitable and unsafe for domestic use, causing different health issues, mostly in immunocompromised persons. Current studies mentioned that these pathogens rarely cause human bacteremia (Chorost et al. 2018; BC Centre for Disease Control 2022). Our findings are consistent with those of Ali et al. (2019); they reported the presence of *B. cereus* and other pathogenic bacteria in spring water. Similarly, another group declared that the *Chutron* hot spring is suitable for bathing but unfit for drinking due to high fluorine content and environmental bacteria (Farhat et al. 2021). Unfortunately, we were unable to identify a few bacteria through MALDI‐TOF MS. It could be due to the absence of reference spectra of novel environmental bacteria, because MALDI‐TOF MS systems are often used for the identification of clinical samples. Our results are consistent with previous reports (Deng et al. 2014; Timperio et al. 2017).

It is believed that taking a bath (balneotherapy) in hot springs reduces pain, skin infections, and other issues in joints (Farhat et al. 2021). It could be associated with the mineral contents or metabolic secretions of microorganisms surviving in these springs. A total of *n* = 7 isolates were pigment‐producing. The discovery of pigment‐producing bacterial isolates holds considerable potential for biotechnological applications. These isolates are promising candidates for producing valuable secondary metabolites, which could have a range of beneficial properties. Specifically, these metabolites may exhibit antioxidant and antimicrobial activities, serve as natural additives or color intensifiers in food and cosmetic industries, and potentially possess anticancer properties, offering new avenues for therapeutic applications. This aligns with findings from a previous research study conducted by Qayyum et al. (2020), which highlighted similar prospects for pigment‐producing bacterial strains (Qayyum et al. 2020). The previously reported literature revealed that Psychrophiles and Mesophiles in the water springs of Pakistan have the potential to yield different bioactive compounds and enzymes for industrial usage (Afridi et al. 2020; Aziz et al. 2019). Similarly, many studies reported that thermophiles could produce hydrolytic enzymes (Kambourova 2018; Zahoor et al. 2016).

## 5 Conclusion

The importance of assessing the microbial diversity in natural water sources, particularly in remote areas like *Arincho* and *Chutron* in Shigar, Gilgit Baltistan, is highlighted in the study. The identification of diverse bacterial strains, including opportunistic pathogens, highlights the potential health risks linked with these springs. The domination of species such as *Brevundimonas* in *Arincho* and *Acinetobacter* in *Chutron* suggests specific microbial compositions unique to each spring. The presence of human‐associated microflora further emphasizes the possible contamination from external sources. The presence of pathogenic bacteria in these water springs could lead to serious health concerns among immunocompromised and elderly persons. On a positive note, these springs also provide a habitat for diverse microbes like pigmented bacteria. These findings underscore the need for stringent water quality monitoring and appropriate measures to ensure the safety of drinking water, especially for vulnerable populations such as immunocompromised and elderly individuals. Continued surveillance and research are crucial for understanding and mitigating the health risks posed by microbial contamination in natural resources of water. In addition, the regulatory authorities should educate natives about contamination and future concerns associated with the use of natural springs. The quality of water sources should be assessed and monitored regularly to provide safe and pure water for the local community and tourists.

## Author Contributions

**Rizwan Abbas:** conceptualization, writing – original draft, and methodology. **Muhammad Haris:** writing – original draft, and methodology. **Sidra Rahman:** writing – review and editing, methodology, and formal analysis. **Syed Ahsan Shahid:** writing – review and editing, validation. **Tasbiha Gul:** investigation, and visualization. **Ayesha Farooq:** investigation, and resources. **Alaa Ismail** and **Gaber El‐Saber Batiha:** funding acquisition, project administration. **Modi O. Alotaibi:** funding acquisition, project administration, reviewed and approved the final version of the manuscript. **Muhammad Ali:** conceptualization, writing – review and editing, supervision, and resources.

## Ethics Statement

The authors have nothing to report.

## Conflicts of Interest

The authors declare no conflicts of interest.

## Data Availability

The data will be available from the corresponding author upon reasonable request.
